# Mathematical modeling of ovine footrot in the UK: the effect of *Dichelobacter nodosus* and *Fusobacterium necrophorum* on the disease dynamics

**DOI:** 10.1016/j.epidem.2017.04.001

**Published:** 2017-12

**Authors:** Jolene Atia, Emma Monaghan, Jasmeet Kaler, Kevin Purdy, Laura Green, Matt Keeling

**Affiliations:** a School of Life Sciences, University of Warwick, Coventry CV4 7AL, UK; b School of Veterinary Medicine and Science, University of Nottingham, Leicestershire LE12 5RD, UK

**Keywords:** Footrot, MCMC, *Dichelobacter nodosus*, Bayesian, *Fusobacterium necrophorum*

## Abstract

*Dichelobacter nodosus* is a virulent, invasive, anaerobic bacterium that is believed to be the causative agent of ovine footrot, an infectious bacterial disease of sheep that causes lameness. Another anaerobe, *Fusobacterium necrophorum*, has been intimately linked with the disease occurrence and severity. Here we examine data from a longitudinal study of footrot on one UK farm, including quantitative PCR (qPCR) estimates of bacterial load of *D. nodosus* and *F. necrophorum*. The data is at foot level; all feet were monitored for five weeks assessing disease severity (healthy, interdigital dermatitis (ID), or severe footrot (SFR)) and bacterial load (number of bacteria/swab). We investigate the role of *D. nodosus* and *F. necrophorum* in the progress of the disease using a continuous-time Markov model with 12 different states characterising the foot. The transition rates between the adjacent states are the (34) model parameters, these are determined using Metropolis Hasting MCMC. Our aim is to determine the predictive relationship between past and future *D. nodosus* and *F. necrophorum* load and disease states. We demonstrate a high level of predictive accuracy at the population level for the *D. nodosus* model, although the dynamics of individual feet is highly stochastic. However, we note that this predictive accuracy at population level is only high in more diseased states for *F. necrophorum* model. This supports our hypothesis that *D. nodosus* load and status of the foot work in combination to give rise to severe footrot and lameness, and that *D. nodosus* load plays the primary role in the initiation and progression of footrot, while *F. necrophorum* load rather increases disease severity of SFR.

## Highlights

• Investigate the role of *D. nodosus* and *F. necrophorum* in the progression of ovine FR.
• Markovian model developed using bacterial load and disease severity.
• The model generates probabilistic forecasts one week ahead.
• All 34 rates between the 12 states of an individual foot are time homogeneous.
• Results suggest primary role of *D. nodosus* in the initiation and progression of footrot.
• Results suggest a secondary role of *F. necrophorum* only in severely diseased feet.

## 1 Introduction

In the UK, footrot is an endemic infectious disease of sheep. It is present in >90% flocks and causes 80% of foot lameness (Kaler and Green, 2008); approximately 5% of the national flock of 16 million ewes are lame at any one time (Winter et al., 2015). Lameness has considerable impact on animal welfare causing pain, discomfort, and weight loss with consequential reduction in productivity through reduced numbers of lambs per ewe and reduced growth rates in affected lambs (Wassink et al., 2010). The economic impact is estimated to be £25–£80 million per year (Nieuwhof and Bishop, 2005, Wassink et al., 2010). Interdigital dermatitis (ID) and severe footrot (SFR) are two pathological presentations of this disease. The clinical presentation of ID is inflammation of the interdigital skin whilst SFR presents as separation of the hoof horn from the sensitive tissue of the foot (Winter, 2008).

The primary aetiological agent of footrot is *Dichelobacter nodosus*, an anaerobic bacterium that can be detected on the feet of healthy, ID, and SFR feet (Moore et al., 2005, Bennett et al., 2009, Witcomb et al., 2014). The load of *D. nodosus* is higher on feet with ID and SFR than in healthy feet (Witcomb et al., 2014). All sheep appear to be susceptible to *D. nodosus* and transmission occurs via the surface on which the animals are kept (pasture or pen). If the skin of the foot is damaged or wet, *D. nodosus* can invade the epidermis and causes disease (Beveridge, 1941). Climate is highly influential in the duration of infectiousness of *D. nodosus* off the host (the lifespan of the bacteria on pasture or in soil when infection can pass to another host) with damp, warm conditions aiding spread of disease (Whittington, 1995, Green and George, 2008). *D. nodosus* survives in warm and damp conditions off the host but cannot be transmitted between sheep in very hot and dry conditions. *Fusobacterium necrophorum*, another anaerobic bacterium, has also been associated with both ID and SFR. It is also detected on healthy, ID, and SFR feet but only detected at a higher loads in feet with SFR (Witcomb et al., 2014), which suggests a secondary or opportunistic role. Hence *F. necrophorum* appears to enhance the disease severity rather than initiate the disease (Beveridge, 1941, Witcomb et al., 2014, Clifton and Green, 2016).

The primary aim of the present study is to quantify the dynamic interaction between bacterial load and disease progression by the development of a mechanistic model; such an approach allows us to speculate on cause and effect more effectively than with a purely statistical model, so can be used to further explore the dynamics of footrot. The model will allow us to establish the relationship between bacterial load and disease progression, and whether a knowledge of the past and future disease states can determine the current load or vice versa.

## 2 Data aquisition

**Fig. 1:**
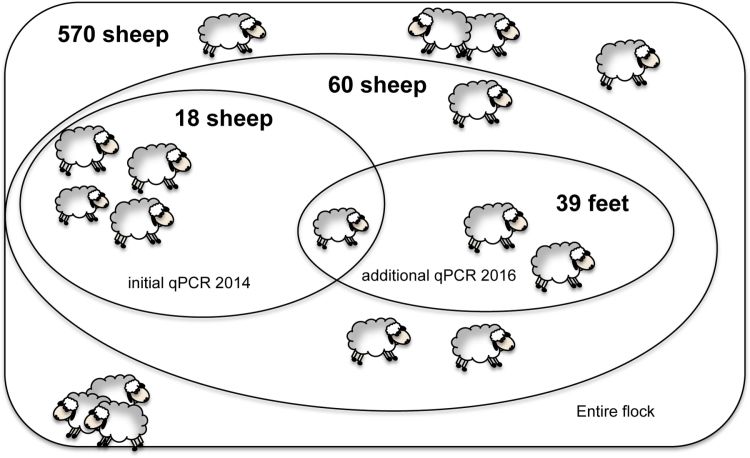
Study population. The study flock consists of 570 sheep. A subset of 60 sheep was selected for monitoring (Kaler, 2011). 18 sheep were subsequently selected for the 2014 study (Witcomb et al., 2014) (labelled as “initial qPCR 2014”) and qPCR was performed to investigate the associations between bacterial load and footrot. Additional foot swabs of 39 feet are selected from the remaining 42 sheep and analysed for *D. nodosus* load using qPCR (labelled as “additional qPCR 2016”), with 4 samples repeated from the initial 18 sheep to test consistency between the 2014 and 2016 qPCR results.

The data used in this paper is from a subset of data from an 18 month longitudinal study of footrot in England in a flock of 570 ewes (Wassink et al., 2010), see Fig. 1. Footrot had been present in the flock for more than 20 years and the average prevalence of lameness caused by footrot was 6–8% (Wassink et al., 2010). From the 570 sheep, 60 were selected and examined each week for 5 consecutive weeks (Kaler, 2011). Each week, the feet were examined and given a disease severity score. The interdigital skin of each foot was swabbed (see Kaler, 2011, Witcomb et al., 2014 for standardised methodology) and swabs were stored in a transport buffer at −80 °C. For the initial part of this study, 18/60 sheep were selected: 3 with no signs of disease throughout the 5-week study; 7 with ID but no SFR; and 8 with at least one foot with SFR (Witcomb et al., 2014). qPCR was used to estimate the loads of *D. nodosus* and *F. necrophorum* (targeting the single copy number of genes rpoD in *D. nodosus*, and rpoB in *F. necrophorum*, in swabs from all feet of the 18 sheep for all 5 weeks). The detailed disease severity scoring and *D. nodosus* and *F. necrophorum* loads used in the previous studies are presented in the supplementary materials.

The initial data therefore comprise a total of 360 observations of 72 feet from 18 sheep. This is characterised by the following set of information: sheep identity, week (from 1 to 5), foot (left front (LF), right front (RF), left hind (LH), right hind (RH)), ID score for each foot, SFR score for each foot (an ID/SFR score of 0 indicates healthy state), *D. nodosus* load on each foot (recorded as number of *D. nodosus* rpoD detected per swab), and *F. necrophorum* load (recorded as number of rpoB detected per swab). The data for the 60 sheep as well as the subset of 18 sheep are shown in the Supplementary Material (data).

### 2.1 Additional analysis of *D. nodosus* load

To assess the predictive power of our model, an additional 200 swab samples were analysed by qPCR. Four samples were repeats from the initial 18 sheep (Witcomb et al., 2014) and the rest were selected from the remaining 42 sheep from the original study of 60 sheep (Kaler, 2011). Individual feet, with the greatest variation in disease severity were selected (see Supplementary Material). The qPCR was performed as before (Witcomb et al., 2014).

## 3 Model formulation

The data for each foot is extremely high-dimensional (with 12 possible states), with a load for the two bacterial species and two scores: one for ID and one for SFR. This high dimensionality precludes the use of complete state-based modelling. We therefore aggregate the values for LOAD (*D. nodosus* bacterial load) and SCORE (disease severity score) for modelling purposes. For LOAD, we use three values (0, 1, and 2): 0 for <10^4^ bacteria/swab, 1 for between 10^4^ and 10^5^ bacteria/swab, and 2 for ≥10^5^ bacteria/swab. We note in Witcomb et al. (2014), our lowest LOAD was partitioned into undetectable bacterial load and detectable load <10^4^ bacteria/swab. We believe our aggregation of these two LOAD is justifiable in terms of both practical detection difficulties and mathematical simplicity. For SCORE, we use four distinct values: 0 for healthy, 1 and 2 for ID and severe ID respectively, and 3 for any SFR irrespective of its ID score. This simplification can be justified by the difficulty of characterising the severity of SFR, but the clear distinction between ID and SRF. Throughout we have made the simplifying assumption that each foot acts independently, ignoring the weak positive correlation presumably because of spatial contamination and the small negative correlations that can arise due to interactions between infection and the host's immune response. Increasing our state space to all four feet on each sheep is infeasible. This simplified model acts as a first attempt to capture the individual-level dynamics of footrot in a mechanistic manner. Finally, we remove from the data 10 feet that had an unexpected behaviour. These feet were healthy (SCORE = 0) through all the 5 weeks of the study but the LOAD was observed to jump from low to high and back again. These fluctuations could be a result of a temporary contamination of the foot where *D. nodosus* does not become established, e.g., because the foot is not damaged) and LOAD reduces at the following observation because disease does not occur.

**Fig. 2:**
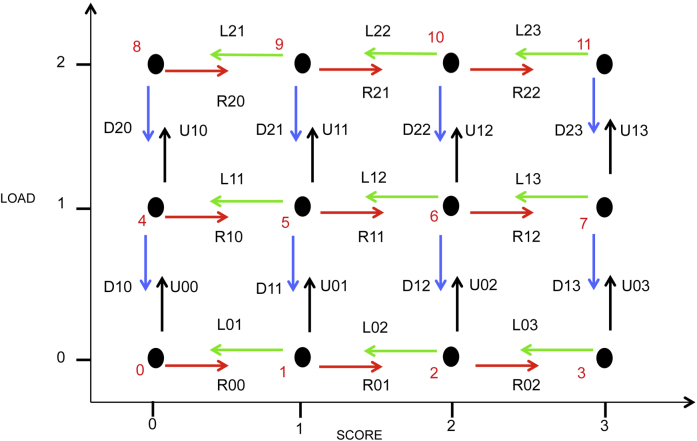
State transition diagram of the Markov model at foot level. The diagram shows the 12 possible state of the foot with different values for LOAD and SCORE. L_*ls*_, R_*ls*_, U_*ls*_, D_*ls*_ are the transition rates going out of the state *ls*, where *l* and *s* are the corresponding LOAD and SCORE, respectively. Numbers in red: the combined score = 4 * LOAD + SCORE. Colours of arrows represent the directions of the transitions. (For interpretation of the references to colour in this figure legend, the reader is referred to the web version of this article.)

From this dataset we develop a model at foot level using a continuous-time Markov model to describe the disease dynamics and the effect of *D. nodosus* on disease severity. We adopt a multistate model with 12 states describing the possible states of the foot in terms of SCORE (four states) and LOAD (three states) (Fig. 2). Rather than work with two-dimensional characterisation, we simplify and use a one-dimensional quantity, which we denote as *Comb*:

(1) Comb=SCORE+4*LOAD.

At any given point in time an individual foot *f* can be in one of 12 states *S* ∈ {0, 1, 2, …, 11} and we have repeated measurements for five weekly time points. We analyse the continuous-time Markov chain using the generator matrix **Q**_*θ*_, where *θ* is explicitly written to acknowledge the parameters within the matrix. The terms in **Q** are the rates of transition (so that **Q**_*θ*_(*i*, *j*) is the rate of transition from state *i* to state *j*), while the diagonals refer to the rate of leaving a given state; hence the rows of **Q**_*θ*_ sum to zero. Under the assumption of continuous time dynamics only transitions between adjacent states are possible (Fig. 2); for instance it is impossible to move from LOAD of 0 to LOAD of 2 without passing through a LOAD of 1. This reduces the number of parameters that determine the matrix *Q*_*θ*_ from 132 (=12 × 11) if all transitions are possible to just 34.

Our data are not in continuous time but are weekly observations (the time of transition from a healthy state to SFR state is generally >7 days Kaler, 2011). It is therefore necessary to transform the continuous time rate matrix **Q**_*θ*_ to a discrete time weekly probability matrix **Z**_*θ*_ Keeling and Ross, 2008:

(2) $${\text{Z}}_{\theta }=\exp{\text{Q}}_{\theta },$$

which depends on the 34 rate parameters *θ* between the neighbouring states. The parameters are denoted L_*ls*_, R_*ls*_, U_*ls*_, D_*ls*_, denoting the weekly leftward, rightward, upward, and downward transition rates between the adjacent states, respectively, where *l* and *s* are the LOAD and SCORE corresponding to the state from which the parameters are moving (Fig. 2). We note that although **Q**_*θ*_ only permits transitions between adjacent states, in the discrete time system (**Z**_*θ*_), all transitions are present yet many have extremely low probabilities. See Keeling and Ross (2008) for more information on methodology.

## 4 MCMC

**Fig. 3:**
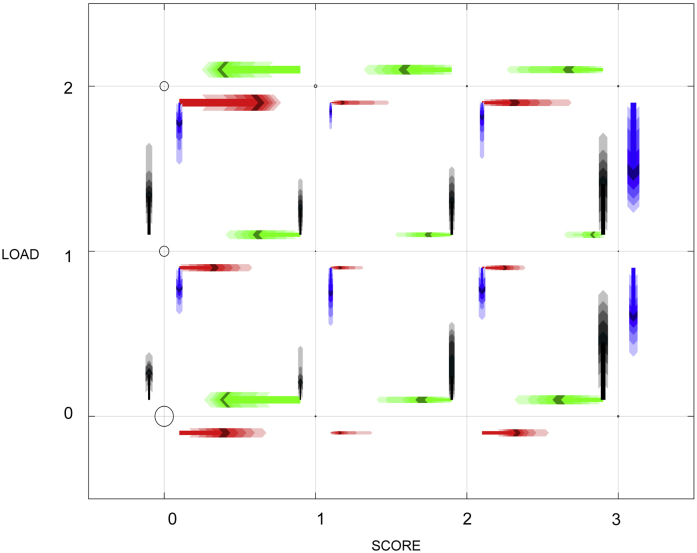
Diagram of the Markov model at foot level showing the probabilities of moving from each state. The diagram shows the results of the MCMC scheme run. Blurred coloured (representing the direction) arrows (different thickness and length) are used to represent the probability that a foot will move from a state to another in a given direction during one week time interval. Circles: the area is a representation of the number of data points in each state. (For interpretation of the references to colour in this figure legend, the reader is referred to the web version of this article.)

We use Bayesian Metropolis–Hasting MCMC to estimate the parameters of the model (Metropolis et al., 1953, Hasting, 1970). The MCMC algorithm provides an efficient method of determining plausible sets of parameters (the posterior) given the likelihood of observing the data. MCMC has considerable advantages when the parameter space is high dimensional and the likelihood is rapidly computed. The likelihood is computed with reference to the discrete time transition matrix

(3) $$L(\theta )=\prod_{f}\prod_{t=1}^{4}{\text{Z}}_{\theta }(S_{f}^{t},S_{f}^{t+1}),$$

where *t* sums over the weeks of the study (capturing the four changes in states), and *f* sums over all feet in the study. In the Bayesian framework it is important to specify a prior, which reflects belief in the unknown parameters. Here, due to limited information, we choose exponential priors on the weekly rates; for the majority of rates we use relatively uninformative exponential priors with a mean of 5, but for transitions from healthy states (SCORE = 0), we use a mean of 1 reflecting our belief that disease (and recovery) is relatively rare. Throughout our proposal parameters are normally distributed about the current set of parameters. We allow the MCMC scheme to run for 2 × 10^6^ iterations, with the first 5 × 10^5^ iterations treated as a burn-in period and ignored (for more information on the MCMC scheme see Supplementary Material). The results of running the MCMC are summarised in Fig. 3. The trace plots of individual parameters indicate that many of the MCMC chains are well mixed and appear to explore the parameter space thoroughly. However, some of the parameters chains do not mix well and a high correlation between some parameters was observed (Figs. S1, S2, S3, and S4 – Supplementary Material).

We used blurred arrows to describe the results of the MCMC showing both means and posterior parameter variation associated with the transition rates (Fig. 3). The arrows represent the probability that a foot will leave a state *S* in a given direction during a 1-week time interval; thicker and longer arrows indicate a higher probability. The colour of the arrow represents the direction as in Fig. 2 (i.e., increasing or decreasing LOAD or SCORE). We note that there are some predominant motions to the most likely transitions (larger arrows), such as a change from low LOAD to a higher LOAD followed by a move to a higher SCORE, although this is subject to considerable stochastic uncertainty.

## 5 Eigenvectors and sensitivity analysis

**Fig. 4:**
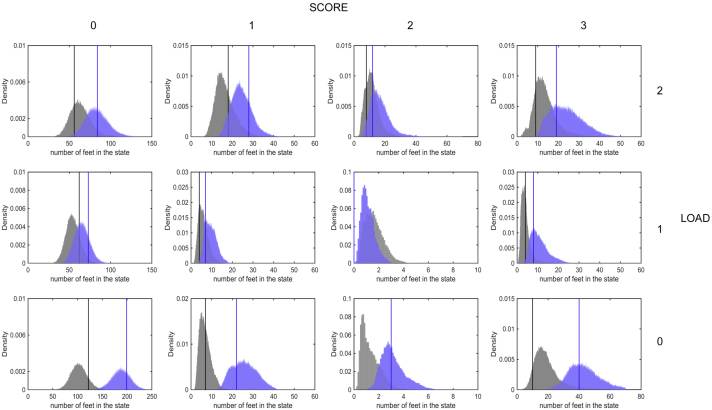
Probability distributions of the eigenvectors. The probability distribution of eigenvectors showing the long-term proportion of time spent in each state; each sample of the MCMC gives rise to an eigenvalue and we use the entire chain to generate these posterior distributions. The vertical lines correspond to the observed distribution across all feet. The results shown in grey correspond to the finding of the initial 18 sheep; the results in blue are for the 18 sheep with the additional qPCR 200 samples. (For interpretation of the references to colour in this figure legend, the reader is referred to the web version of this article.)

We wish to assess this modelling approach by using the available data in an alternative form. The eigenvector associated with the dominant eigenvalue of *Z*_*θ*_ (which will be zero), gives the long-term probability distribution of the combined state for a randomly chosen foot. We therefore compare the dominant eigenvectors from the posterior set of *Z*_*θ*_ (i.e., with parameters sampled from the MCMC chain), with the observed frequency of combined states (Fig. 4).

Despite the fact that the parameters are fit only to the observed transitions, the eigenvector (and hence the long-term distribution) shows close agreement with the frequency of each observed state. Healthy feet (LOAD 0, SCORE 0) dominate in both observation (122 healthy feet) and long-term prediction (mean 102, 95% prediction interval = 73–133); in addition SCORE 0 and LOAD 2 is also common in both predictions (mean 62, 95% prediction interval = 40–85) and data (56 feet), while SCORE 2 and LOAD 0 or 1 are vanishingly rare (Fig. 4 grey). This agreement between observations and long-term dynamics provides an independent validation of the model fit, beyond the likelihood used to generate the model fitting.

To test the sensitivity of these results to each of the parameters, we also compute the change of dominant eigenvectors with a change to each of the 34 transition parameters. This is achieved by first setting all of the parameter values to their median; we then compute the eigenvector from the corresponding probability transition matrix (**Z**_*θ*_). We next increase the values of each parameter by 10% from the median and recalculate the dominant eigenvector. We quantify the rate of change of the eigenvectors against each parameter using the root mean square change (RMS) of the eigenvectors (rounded to the nearest thousandth) (Table 2). We note that the parameters that have the highest impact on the eigenvectors are the same parameters that are better determined by our MCMC scheme (parameters in bold in Table 2); the model has greater sensitivity to those parameters.

## 6 Analysis of predicting individual feet and model validation

**Fig. 5:**
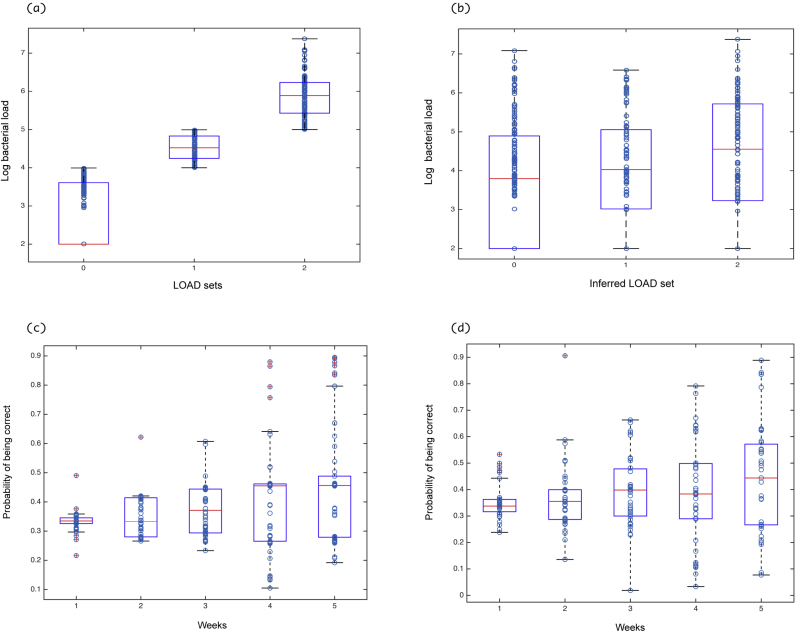
Predictive ability of the model. (a) Box plot and scatter plot for the true LOAD vs the log of the bacterial count for the 18 sheep, (b) box plot and scatter plot for the inferred LOAD vs the log of the bacterial count for the 18 sheep, (c) box plot and scatter plot showing the predictive ability of the model in time for the 18 sheep, (d) box plot and scatter plot showing the predictive ability of the model in time for the new swabs with 100-fold modification factor.

To test the predictive ability of the model, we infer the LOAD for the 18 sheep simply using their score data (Witcomb et al., 2014). This has a clear practical applications for future data collection because score is easier and cheaper to measure than bacterial load. In particular we use the recorded SCOREs across the five weeks to infer the LOADs most likely to have generated the observed transitions; for instance a change from low to high SCORE is unlikely without high LOAD. Given the estimated parameters (and hence the transition matrix), we can compute the likelihood of every possible combination of five LOAD values to augment the SCORE values. Fig. 5a and b shows a scatter and a box plot of the real and inferred LOAD of the 18 sheep versus the log of the measured bacterial count (in number of bacteria/swab). Any load recorded as ‘below the limit of detection’ was replaced by 200 bacteria per swab, this is justified as the minimum number of bacteria detected is around 10^3^ bacteria per swab. We note that our inferred LOAD generates a high variability in the log of the bacterial count. This suggests a high stochasticity in the model.

To provide further understanding of this inference, we examine the probability of predicting the correct original LOAD in each week (Fig. 5c and d). Using the probability of every possible combination of five LOAD values corresponding to the recorded SCORE values, we compute the total probability of obtaining the observed LOAD values in each week. We note from the Fig. 5 c that there is an improvement in the predictive ability (an increase in the probability of being correct) with time; the maximum probability is reported in week 5.

To further confirm the reliability of the MCMC model, we analyse additional samples from the original study (Kaler, 2011) using q-PCR (Section 2.1). A problem that we encountered was the age of the samples and the cycles of freezing and thawing over time which may have led to loss of detectable DNA and therefore a lower estimate of bacterial load. Four samples were repeat analyses from the 18 sheep for comparison. We note a 100 fold factor difference between the original 18 sheep and the repeated samples. We analyse the new samples (with 100 fold modification factor) in a similar way to the original 18 sheep, by looking at the probability of predicting the correct LOAD in each week (Fig. 5d). There is a similarity between the prediction ability of the model for the two data sets as shown in Fig. 5. Finally, we re-run the MCMC scheme with both qPCR sets included, then used the eigenvector method to check if there is any improvement in the predictions. Healthy feet (LOAD 0, SCORE 0) again dominate in both observation (122 healthy feet) and long-term prediction (mean 102, 95% prediction interval = 73–133); in addition SCORE 0 and LOAD 2 are also common in both predictions (mean 62, 95% prediction interval = 40–85) and data (56 feet), while SCORE 2 and LOAD 0 or 1 are vanishingly rare (Fig. 4 blue). An increase in the probability of being correct through weeks also holds for the new set of data (Fig. 5d). Consequently, we use the new set of data, with a factor of 100-fold, in conjunction with the data from the 18 sheep to re-run the MCMC Metropolis Hasting to re-estimate the parameters. An improvement in the convergence of many parameters is detected. We also note a refinement in the long-term behaviour in comparison with the density of the observed data.

## 7 The effect of *F. necrophorum* LOAD on SCORE

The MCMC scheme for estimating the parameters associated with the *F. necrophorum* can be readily performed in a similar method as that used for *D. nodosus*. We use the same coding system for the LOAD and SCORE; and in a similar manner we remove the individual 5 weeks data of 3 healthy feet that were observed to fluctuate. We validate the modelling approach by using the eigenvector analysis as described in Section 5. Comparing the dominant eigenvectors distribution from the posterior set of the parameters sampled from the MCMC chain with the observed frequency of the combined states, we note that there is no agreement between the two in the healthy states but there was a consistency among them in the more diseased states (Fig. S5 in supplementary material). We note 154 observed healthy feet (LOAD 0, SCORE 0) and a mean of 138 long-term prediction (95% prediction interval = 132–175), 116 feet with SCORE 0 and LOAD 1 and a prediction mean of 96 (95% prediction interval = 78–114), while for SCORE 3 and LOAD 1, the number of feet is 10 and the prediction mean is 9 (95% prediction interval = 3–14) (Fig. S5).

## 8 Bacterial load inference from disease severity score

As extension to our initial model, we wish to utilise the data from the 42 sheep in the study that did not have a bacterial load assessed by qPCR (Kaler, 2011). Again, we use the recorded SCOREs across the 5 weeks to infer the most likely LOAD to have generated the observed transitions (method shown in Section 6). Sampling randomly according to this likelihood allows us to inflate our data set from the original 18 sheep to the full 60 sheep. This overcomes the sampling biases in the original selection of the 18 sheep. The MCMC scheme is rerun with the 60 sheep giving a total of 1200 recordings of 240 feet from 60 sheep. We iterate this process in order to obtain consistent parameter estimates. The results from this larger inferred data set are shown in Fig. S4 (blue) (Supplementary Material); again we find that there is a very strong agreement between the dominant eigenvector (and hence the expected long-term behaviour) and the observations. However, by including all 60 sheep that were part of the study, we have overcome the initial biases that came from selecting 18 sheep with particular characteristics. We find that by considering all 60 sheep, the rate of transition from *Comb* score 1 to 0, 2 to 6, 6 to 7 and 7 to 11 are substantially increased and from *Comb* score 5 to 1, 6 to 2, 6 to 10, 9 to 10, 10 to 11, and 11 to 10 are decreased and correspondingly the expected proportion in states of *Comb* score 6, 9, and 11 relatively increases (Table 1 shows the combined score in terms of SCORE and LOAD).

**Table 1:** Combined score in terms of SCORE and LOAD.

Comb	SCORE	LOAD
0	0	0
1	1	0
2	2	0
3	3	0
4	0	1
5	1	1
6	2	1
7	3	1
8	0	2
9	1	2
10	2	2
11	3	2

**Table 2:** RMS of the change of the eigenvectors to small changes in each of the parameters.

Parameter ID	Parameter	RMS of change	Transitions $S_{f}^{t}\to S_{f}^{t+1}$^a^
1	**U00**	0.021	(0,0) → (0,1)
2	U01	0.007	(1,0) → (1,1)
3	U02	0.002	(2,0) → (2,1)
4	U03	0.003	(2,0) → (2,1)
5	**U10**	0.016	(0,1) → (0,2)
6	U11	0.007	(1,1) → (1,2)
7	U12	0.002	(2,1) → (2,2)
8	U13	0.003	(3,1) → (3,2)
9	**D10**	0.02	(0,1) → (0,0)
10	**D11**	0.01	(1,1) → (1,0)
11	D12	0.001	(2,1) → (2,0)
12	D13	0.004	(3,1) → (3,0)
13	**D20**	0.016	(0,2) → (0,1)
14	D21	0.007	(1,2) → (1,1)
15	D22	0.002	(2,2) → (2,1)
16	D23	0.003	(3,2) → (3,1)
17	**L01**	0.01	(1,0) → (0,0)
18	L02	0.005	(2,0) → (1,0)
19	L03	0.004	(3,0) → (2,0)
20	L11	0.005	(1,1) → (0,1)
21	L12	0.002	(2,1) → (1,1)
22	L13	0.001	(3,1) → (2,1)
23	**L21**	0.01	(1,2) → (0,2)
24	L22	0.006	(2,2) → (1,2)
25	L23	0.003	(3,2) → (2,2)
26	**R00**	0.01	(0,0) → (2,0)
27	R01	0.005	(1,0) → (2,0)
28	R02	0.004	(2,0) → (3,0)
29	R10	0.006	(0,1) → (1,1)
30	R11	0.002	(1,1) → (2,1)
31	R12	0.002	(2,1) → (3,1)
32	**R20**	0.01	(0,2) → (1,2)
33	R21	0.007	(1,2) → (2,2)
34	R22	0.005	(2,2) → (3,2)

a *f*-individual foot at time *t* with state *S* with (*s*, *l*), *s*-SCORE, *l*-LOAD.

## 9 Discussion

In this work we have explored the relationships between footrot disease severity score and *D. nodosus* bacterial load in a study population of sheep. We have shown that the behaviour of individual feet can be represented by a relatively simple (but high-dimensional) Markov state moment, where the parameters can be inferred with substantial computational efficiency. Despite the pronounced stochasticity at the individual-level, such that we cannot accurately predict the status of any given foot at the next sample, at population level our model captures the observed behaviour. This suggests that this type of Markov model could be highly effective at predicting the likely impact of interventions at the flock-level.

We have developed a full state-based mathematic model, that is matched to data on foot health and bacterial load through Bayesian parameter inference (Fig. 2). The continuous-time model, where all the transitions are between adjacent states, comprises 12 states; each state describes the state of a foot at one point in time in terms of SCORE and LOAD. The 34 parameters are the transition rates between the adjacent states. Our model makes the simplifying assumption that each foot acts independently and therefore ignores the positive effects of local environment and the negative effects of the host immune response. We expect the impact of such terms to be relative minor (second order) to the foot-level dynamics, as they should only come into play once two feet are diseased, which occurs with a relatively low probability (⋍13% of all 18 sheep in the 5 weeks), even under the assumption of independence.

There are a number of obstacles to the use of the MCMC for our data set, specifically convergence problems for some parameters. Many parameter chains are not mixing well (convergence test performed by looking at the MCMC chains); this may be due to the large parameter space and the small data set we use and high correlation between the parameters of interest. However our scheme seems to work well in practice. We compute the eigenvector associated with the probability transition matrix associated with the dominant eigenvalue by sampling from the parameters MCMC chain. This gives the long-term probability distribution of the 12 states for a randomly chosen foot. The distribution of the eigenvectors, which gives the predicted equilibrium level of SCORE and LOAD, is in a broad agreement with the observed data (Fig. 4) despite the model being dependent only on the observed transitions; the frequency of the observed data in each state has not been used in the modelling process. This provides a validation of the model and shows that the model is capturing the population dynamics. In addition, the RMS of change of eigenvectors due to small changes in each of the parameter values indicates that the model is more sensitive to the parameters that are better determined with the scheme (Table 2). We show that although some parameters are unidentifiable by the model, the latter is insensitive to those parameters and that they are not relevant to the dynamics of the disease. For instance, a state of high SCORE (1 or 2) and low LOAD is rare, which implies that the transitions from and to this state are not relevant. It is thus not viable to learn about those parameters even from a complex analysis of the data.

In order to check if the LOAD provides information about the process, we ran the MCMC using only one variable, the SCORE. The resulting state model comprises just 4 states of the foot (SCORE of 0, 1, 2, and 3) and 6 transition rates between adjacent states. We compute the eigenvector distribution of the associated 4×4 transition matrix. In this case, the long-term behaviour was not in agreement with the observed frequency (See figure S6 in Supplementary Material), which suggests that LOAD is affecting the process. This is in agreement with the studies which examined *D. nodosus* and demonstrated that the organism is important in the development and presence of ID and progression of SFR (Beveridge, 1941, Kennan et al., 2001, Kennan et al., 2010, Witcomb et al., 2014, Clifton and Green, 2016).

For *F. necrophorum*, we note that the long-term behaviour is not in agreement with the observed frequency in the healthy states, but consistent with the observed frequencies in the more diseased states (see Fig. S5 in Supplementary Material). This result is consistent with the previous studies where an increase in *F. necrophorum* load is only observed once SFR had developed (Witcomb et al., 2014). This suggests that *F. necrophorum* enhances disease severity rather than having a role of a precursor.

Using the recorded SCOREs across the five weeks, we attempt to infer the most likely LOAD to have generated the observed transitions (method explained in Section 6). To visualise the results of the inference, we plotted the inferred LOAD versus the observed log_10_ of the bacterial load (Fig. 5). The results suggest a high stochasticity in our model; a high variability was observed for each of the LOAD sets. This result is reasonable because the disease is affected by many random external events such as temperature, rain fall (Green and George, 2008, Smith et al., 2014), and pasture (Kaler et al., 2010), and soil type (Muzafar et al., 2015), all of which influence the probability of a foot being damaged and so becoming susceptible to infection. The data available were for only 5 weeks, which is a short period of time given the time to development of SFR is >1 week and frequently >2 weeks (Kaler, 2011). Additionally, this is a relatively short sample, so seasonal variation in temperature and rainfall is not likely to play a role. For longer-term studies we would expect some of the transition rates to depend on climate variables. Also, during this five-week study, there is insufficient change in the bacterial load over time to be able to see its impact on the foot state. It is thus difficult to infer at individual foot level with reasonable confidence (with the current model and the current data set).

We further assess the predictive ability of the model by looking at the probability of inferring the original LOAD correctly in each week. We use a box plot to look at the probability of being correct in each week. We notice an increase in the probability, with the maximum reported in week 5 (Fig. 5c and d). This demonstrates that there is a relationship between LOAD and SCORE, but that it is subject to substantial amounts of stochasticity such that only with multiple observations of the score can the load be predicted, and even then with only moderate levels of accuracy. This does not contrast with previous statistical findings (such as the statistical correlations between load and footrot at the population level reported previously Witcomb et al., 2014), instead it highlights the extreme variability at the level of individual feet.

## 10 Conclusion

The observed dynamics of footrot infection on the ovine foot is inherently complex and stochastic, although previous statistical analysis have detected correlations in the behaviour (Beveridge, 1941, Kennan et al., 2001, Kennan et al., 2010, Moore et al., 2005, Bennet et al., 2009, Witcomb et al., 2014, Witcomb et al., 2015). Here we have developed a relatively simple predictive model, which uses both bacterial load and disease severity to generate probabilistic forecasts one week ahead. This model is Markovian, such that only the status of the foot (in terms of load and disease severity) for the current week is needed to probabilistically predict future dynamics. Given that the observations occur over a short time period when the climate and total bacterial load remain relatively invariant, we have been justified in assuming that all transition rates in the model are time homogeneous. Using *D. nodosus* bacterial load and footrot disease severity score, the Markovian model is a reliable fit to the data, as evidenced by a relatively high likelihood and close agreement between the aggregate data and the predicted long-term steady-state of the model. In contrast, if *F. necrophorum* bacterial load is used instead, we are unable to accurately capture the full dynamics. These mathematical results give support to our belief that while the dynamics of individual feet are highly stochastic, there is a strong underlying pattern that emerges at the flock-level. This pattern is driven by the interaction between *D. nodosus* load and the health of the foot (as indexed by the footrot disease severity score), with *F. necrophorum* only playing a potential role in highly diseased feet. The model developed here opens the possibility of long-term prediction of footrot dynamics at the flock-level and hence the ability to investigate the impact of random or targeted interventions, although additional factors, such as the impact of climate, will need to be incorporated.

## Authors’ contribution

Designed the experiments – L.G., J.K., M.K., K.P. Recruited the farm, data collection and recording from sheep – J.K., L.G. Performed the lab analysis – E.M., K,P. Analysed the data – J.A., M.K. and contributed to academic discussion – L.G., K.P., J.K. Wrote the paper – J.A., M.K., L.G., K.P., J.K.

## Funding

Funded by grant from BBSRC (ref BB/M012980).
