# Supplementary material for: Mathematical modeling of ovine footrot in the UK: the effect of Dichelobacter nodosus and Fusobacterium necrophorum on the disease dynamics
Source: Epidemics. 2017 Dec;21:13–20. doi: 10.1016/j.epidem.2017.04.001 (PMC5729202; doi:10.1016/j.epidem.2017.04.001)
Supplement: Supplementary file 1 [file mmc1.pdf]

Supplementary Material

Disease severity scoring and load

In Kaler et al [1], 60 ewes were selected from a flock of 570 ewes. For 5 weeks, all 4 feet of all 60 ewes were examined and swabbed each week and given a disease severity score as follows; score 0 for healthy, score 1 for mild ID, score 2,3 for ID, and score 4 for severe ID. A separate SFR score from 1 to 12, based on SFR severity using a defined scoring system [2, 3], was given to each foot. The swabs were stored and used in the next study [4]. Witcomb et al [4] selected 18 sheep from the 60 sheep in the previous study and used specific qPCR to investigate the change in load of *D.nodosus* and *F.necrophorum* in the sheep's feet. *D.nodosus* and *F.necrophorum* loads were summarised as score 0 for below the limit of detection, score 1 for $< 10^4$, score 2 for $10^4 - 10^5$, score 3 for $10^5 - 10^6$, score 4 for $\geq 10^6$ copies per swab (see supplementary materials-data), but they used log load in their calculations. The scoring used in all the studies of interest for the disease severity and the bacterial load are shown in tables S1 and S2, respectively.

**Study**	**variable**	**score given for each foot**							
**Kaler et al [1]**	FR	0					$1 \rightarrow 3$	$4 \rightarrow 6$	$7 \rightarrow 12$
	ID	0	1	2	3	4	$0 \rightarrow 4$	$0 \rightarrow 4$	$0 \rightarrow 4$
**Witcomb et al (data) [4]**	FR	0					1	2	3
	ID	0	1	2	3	4	$0 \rightarrow 4$	$0 \rightarrow 4$	$0 \rightarrow 4$
**current study**	ID/FR	0		1	2		3		

Table S1. Disease Severity Score

**Study**	**variable**	**score given for each foot**				
**Witcomb et al (data) [4]**	number of bacteria/swab	undetected	$< 10^4$	$10^4 - 10^5$	$10^5 - 10^6$	$\geq 10^6$
	Load	0	1	2	3	4
**current study**	LOAD	0		1	2	

Table S2. Bacterial load

MCMC

The MCMC algorithm fits a statistical model to a data set by generating a random sample from the target distribution (the posterior). The generated sample serves as an approximation to the probability distribution for further inference. For our model, the unknown parameters are the transition rates between

adjacent states, giving us 34 parameters to estimate. We set up the initial model parameters and calculate the initial likelihood. The likelihood is capturing the state transitions of the foot by picking up terms from the probability matrix according to the data. We alter all the parameters slightly with each iteration recalculating the likelihood and the prior, comparing the new posterior probability to the old one, and choosing whether to accept the new parameter values. Acceptance condition is:

$$\frac{P_{new}}{P_{old}} > u \ , \tag{1}$$

where $u \backsim U[0,1]$. The new set of parameter values will be accepted if the new posterior probability is higher than the old one. If the old one is higher, then they are accepted with a small probability.

The likelihood is computed with reference to the discrete time transition matrix. The posterior probability proportional formula is:

$$P \propto \prod_{f} \prod_{t=1}^{T-1} z_\theta(S_f^t, S_f^{t+1}). \prod_{k=1}^{a} \exp\left(-\theta_k/p\right) . \tag{2}$$

T is the number of time points, and a is the number of parameters. It is more useful to use the log likelihoods because the values tend to be extremely low. For each parameter, we sample randomly from a normal distribution and add this value to the old parameter value;

$$\theta_{k_{new}} = \theta_{k_{old}} + N(0, \sigma_{\theta_k}) \ , \tag{3}$$

where the variance σ_θ is chosen to be relatively small values compared to the initial conditions of the parameters, k refers to the different parameters ($k \in \{1, 2, \ \cdots 34\}$). A condition is set for the values of the parameters to be positive. A simple prior is used for each of the parameters (equation (2)). We have no previous knowledge of the likely parameter values, hence we assume the prior to be an exponential distributions for all parameters. We vary p according to our understanding of each of those parameters; for instance the rate parameters moving out of the healthy states tend to be slower owing to the fact that many stationary feet were found in the healthy states. Consequently we choose a tighter prior with $p = 1$, for the other parameters we take $p = 5$. The burn-in period includes the first $5{\times}10^5$ iterations. Figures S1, S2, S3, and S4 show the results of the MCMC scheme probability densities of the estimated parameters.

Figures

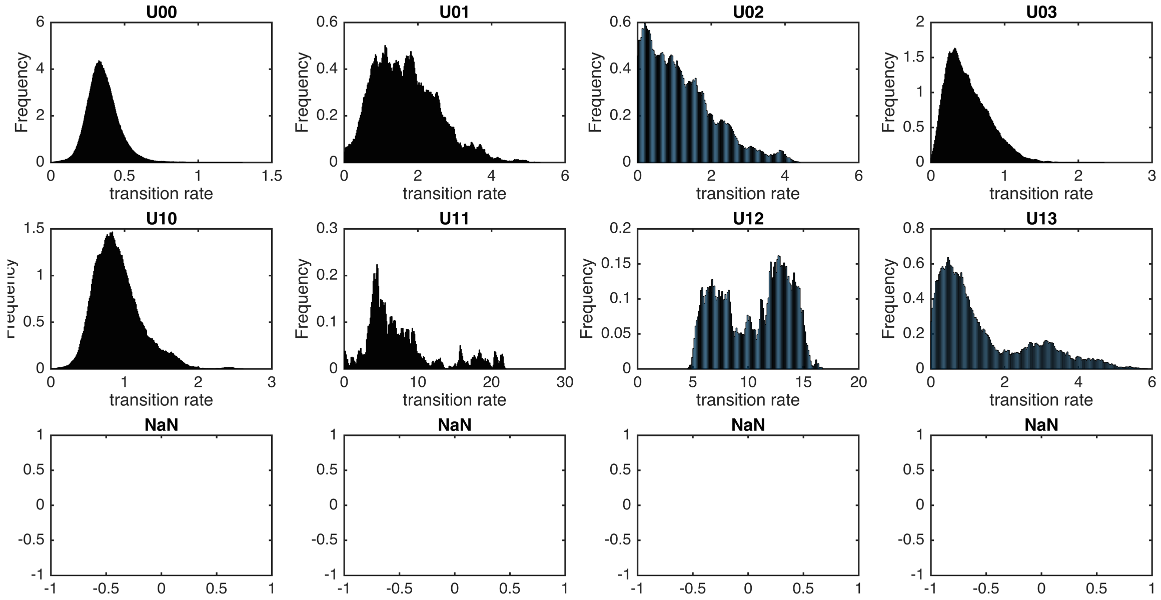

Figure S1. Upward transition rates
The posterior distribution of the upward transition rates U_{ls} going out of the state ls, where l and s are the corresponding LOAD and SCORE, resulting from the MCMC run for 2×10^6 iterations (with a burn-in period of 5×10^5 steps) from the 18 sheep.

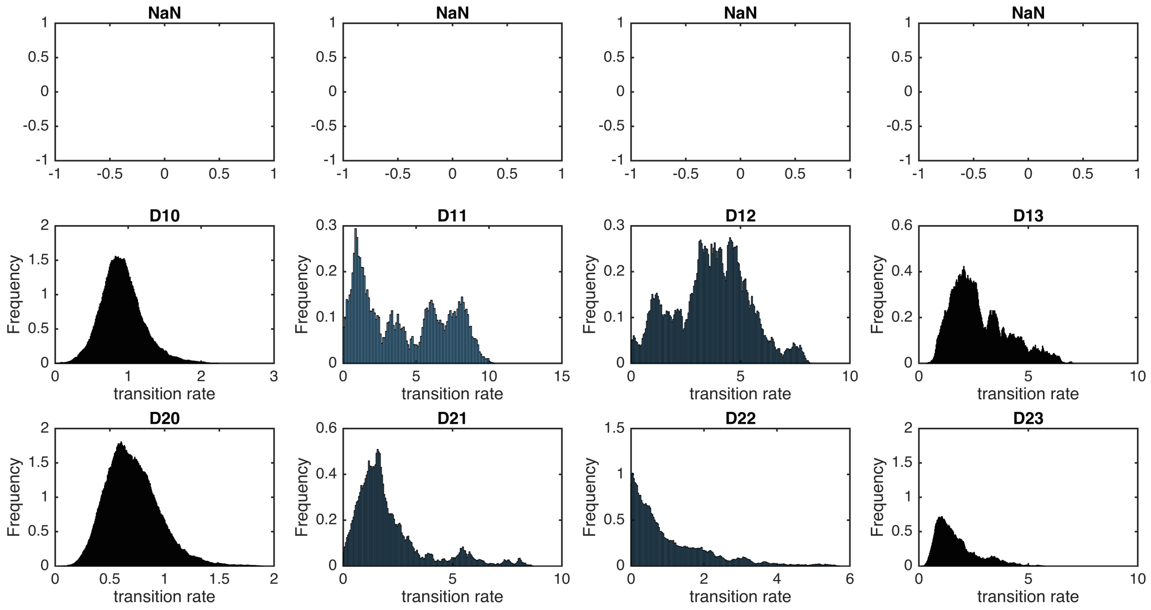

Figure S2. Downward transition rates
The posterior distribution of the downward transition rates D_{ls} going out of the state ls, where l and s are the corresponding LOAD and SCORE, resulting from the MCMC run for 2×10^6 iterations (with a burn-in period of 5×10^5 steps) from the 18 sheep.

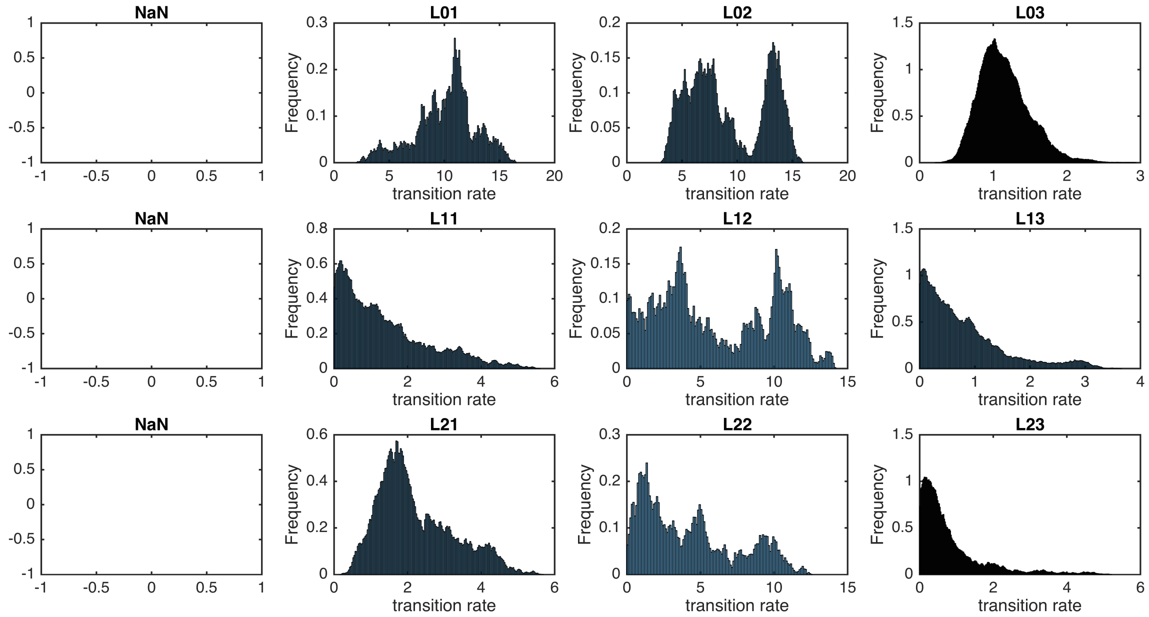

Figure S3. Left transition rates
The posterior distribution of the left transition rates L_{ls} going out of the state ls, where l and s are the corresponding LOAD and SCORE, resulting from the MCMC run for 2×10^6 iterations (with a burn-in period of 5×10^5 steps) from the 18 sheep.

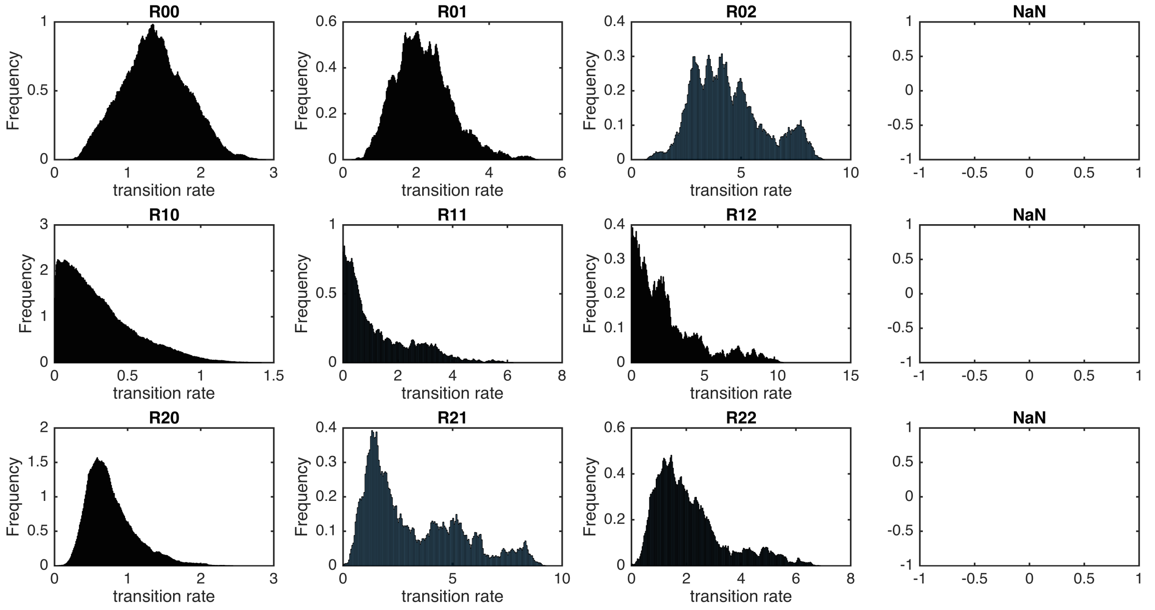

Figure S4. Right transition rates
The posterior distribution of the right transition rates R_{ls} going out of the state ls, where l and s are the corresponding LOAD and SCORE, resulting from the MCMC run for 2×10^6 iterations (with a burn-in period of 5×10^5 steps) from the 18 sheep.

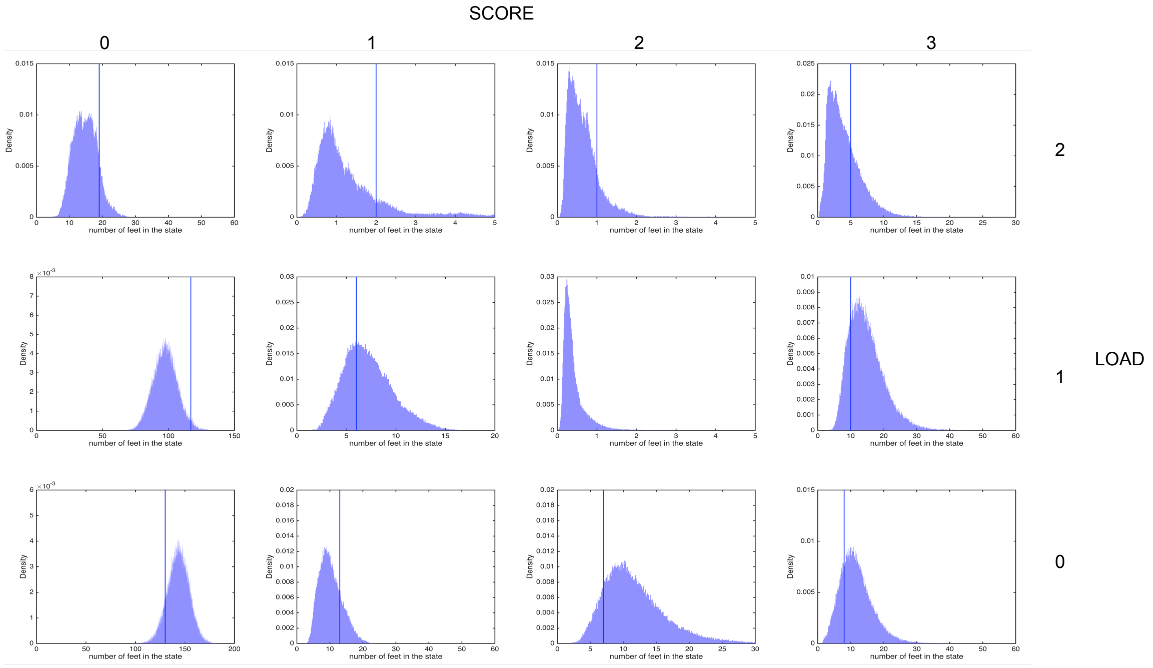

Figure S5. Probability distributions of the eigenvectors for the *F.necrophorum*
The probability distribution shows the long-term proportion of the time spent in each state. Sampling is from MCMC run from the 18 sheep. The vertical line indicates the observed number of feet in each state.

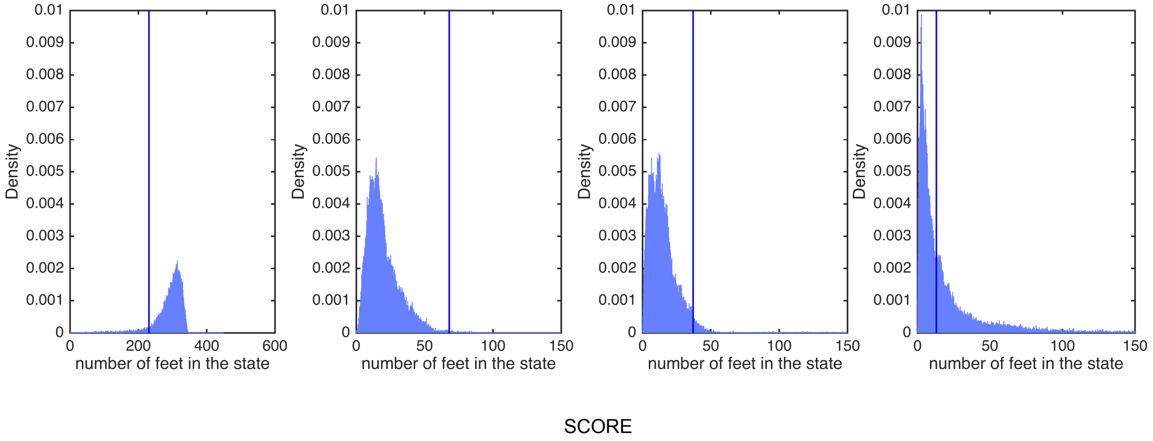

Figure S6. Probability distributions of the eigenvectors with SCORE being the one variable
The probability distribution shows the long-term proportion of the time spent in each state. Sampling is from MCMC run with only SCORE as a variable from the 18 sheep. The vertical line indicates the observed number of feet in each state.

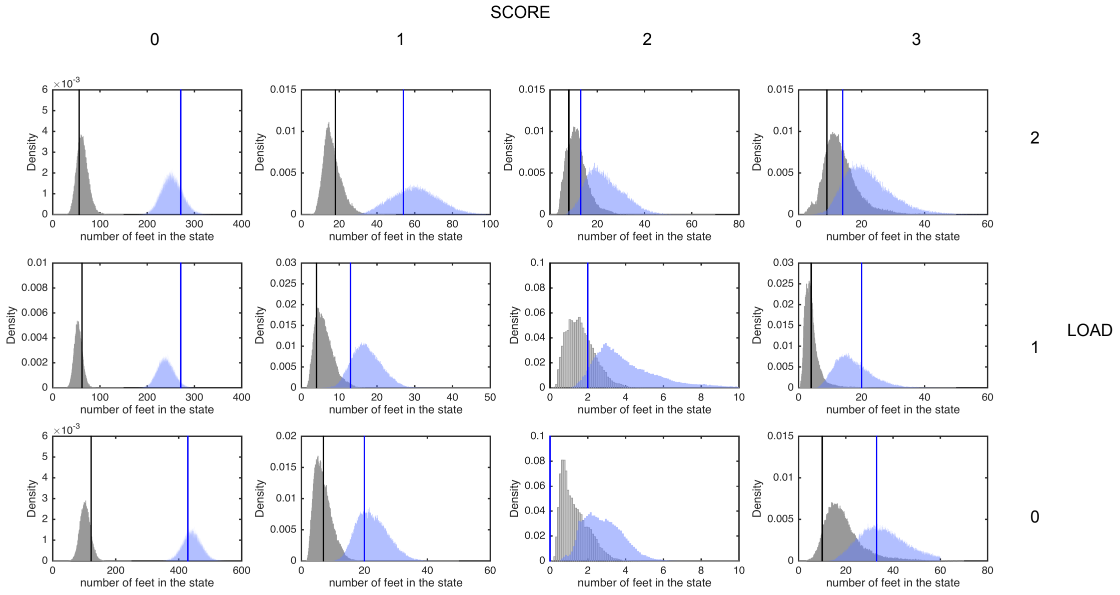

Figure S7. Probability distributions of the eigenvectors for the *D.nodosus*
The probability distribution of eigenvectors showing the long-term proportion of time spent in each state; each sample of the MCMC gives rise to an eigenvalue and we use the entire chain to generate these posterior distributions. The vertical lines correspond to the observed distribution across all feet. The results shown in grey correspond to the finding of the initial 18 sheep; the results in blue are for the inferred status of the 60 observed sheep.

References

1. Kaler J, George RN T, Green LE. Why are sheep lame? Temporal Associations between severity of foot lesions and severity of lamenss in 60 sheep [Journal Article]. Anim Welf. 2011;20:433–438.

2. Egerton JR, Roberts DS. Vaccination against ovine foot-rot [Journal Article]. J Comp Path. 1971;81:179–185.

3. Foddai A, Green LE, Mason SA, Kaler J. Evaluating observer agreement of scoring systems for foot integrity and footrot lesions in sheep [Journal Article]. BMC Vet Res. 2012;8:65.

4. Witcomb LA, Green LE, Kaler J, Ul-Hassan A, Calvo-Bado LA, Medley GF, et al. A longitudinal study of the role of *Dichelobacter nodosus* and *Fusobacterium necrophorum* load in initiation and severity of footrot in sheep [Journal Article]. Prev Vet Med. 2014;115(1-2):48–55.
